# Research on the coordination of energy in China’s economic growth

**DOI:** 10.1371/journal.pone.0251824

**Published:** 2021-06-11

**Authors:** Yuanying Chi, Guoqing Bai, Jialin Li, Bin Chen

**Affiliations:** 1 School of Economics and Management, Beijing University of Technology, Beijing, China; 2 China Datang Corporation Ltd., Beijing, China; 3 State Power Investment Group Corporation, Beijing, China; Universidad de Malaga Facultad de Ciencias Economicas y Empresariales, SPAIN

## Abstract

This study uses the improved Cobb-Douglas two-factor production function model to explore the potential relationship between economic growth and energy consumption through the multiple co-integration test on the panel data of China from 1985 to 2018. The results show that there is a positive long-term balance between energy consumption and economic growth: economic growth of 1%, total energy consumption growth of 1.53%, which means that economic growth needs higher energy support in the former short term. At the same time, the error correction term will converge energy consumption to a long-term equilibrium state with an adjustment intensity of 134.59%. From the results of variance decomposition, we can also see that as the number of periods increases, the part of real economic growth explained by energy consumption gradually increases.

## 1. Introduction

After more than 40 years of reform and opening up, with the development of China’s urbanization and industrialization, economy has not only continued to maintain rapid growth. Moreover, China’s dependence on energy is becoming more and more deep, and the amount of energy consumed every year is increasing. However, the gap between energy supply and demand is growing, and the imbalance between energy supply and demand is increasing. At the current stage, coal is the main energy source used in China [1–3]. Although the potential and advantages of new energy are very suitable for future development, the development and utilization of new energy cannot be achieved in a day. In recent decades, China’s economic development has improved significantly, but the comprehensive economic growth pattern and coal-dependent energy consumption structure have increased pollution and environmental damage. Therefore, this article explores energy-GDP relationships and discusses how to use energy efficiently to ensure sustainable development.

It is of great significance to study the relationship between energy consumption and economic growth, whether from a theoretical perspective or from an experience and policy perspective. Therefore, there are a large number of related theories and literature. Relevant research on the relationship between energy consumption and economic growth can be traced back to Kraft, who used the causal test method to examine the correlation data of energy consumption and economic growth in the United States from 1947 to 1974, and concluded that there was a one-way causal relationship between them, made a groundbreaking work [4].

Many scholars have explored the impact of changes in energy intensity and energy efficiency on economic growth. Díaz, A. and Marrero, G. A. et al. explored, using a dynamic panel approach, how changes in energy intensity and the switch to renewables can boost economic growth [5]. Yaw Naminse, E., & Zhuang, J. mainly uses static and dynamic regression, Granger causality and impulse response function to study the relationship between China’s economic growth, energy intensity and carbon dioxide emissions [6]. Mahmood, T., & Ahmad, E. found that European countries have been able to use less energy through their economic growth [7]. Literature studies show that with the improvement of energy intensity and energy efficiency, economic growth is becoming less and less dependent on energy consumption [7–9]. What is the current relationship between energy consumption and economic growth under pressure to reduce carbon emissions?

In subsequent studies, Granger causality test technique and bivariate co-integration test were used to test the relationship between energy consumption and GDP in different countries [10–15]. However, some scholars later found that the common Granger causality test could not distinguish the source of the causality [16, 17]. In the case of cointegration between variables, the Vector Error Correction (VEC) model can distinguish long-term and short-term relationships between variables, and can effectively identify causality Source of relationship [18–20].

In addition, it is generally believed in the literature that due to the excessive simplification of the model in the causality test process, omitting some variables may lead to spurious regression [21]. Therefore, recent research in this field tends to take energy as a basic element of economic activity and incorporate into a pluralistic framework to study the Granger causality between energy consumption and economic growth [22–28].

Judging from the research methods in the literature, they tend to use co-integration tests, the vector autoregressive (VAR) or VEC method to make judgments on energy-GDP long-term relationship and causal direction [29–35]. At the same time, it is also found that when the selected region and time are different, the conclusions obtained are also quite different [26–28, 36–38]. This makes the research results of the relationship between the economic growth and energy consumption in a certain area very specific and reference. When exploring the relationship between energy and economy, some articles only select the two variable sequences of energy and economy [39–41]. However, it is obvious that economic changes are not only related to energy, which makes it difficult to identify the fundamental source of the causal relationship between energy and economy.

In recent years, with the acceleration of industrialization and urbanization in China, the impact of sustained economic growth on energy consumption has been increasingly intensified. Moreover, the implementation of energy consumption and carbon emission reduction policies has a greater impact on economic growth. Many scholars have added energy as an influencing factor to the Cobb-Douglas two-factor production function model [42, 43]. However, the traditional neoclassical economic growth model only takes energy input as an intermediate factor to investigate the impact of energy consumption on economic growth [44–46]. Most scholars regard the role of energy in production as a neutral factor, and labor and capital as the main basic factors [42, 44, 45, 47]. As a result, the contribution of energy to production has been marginalized. Therefore, with the deepening of China’s industrialization and the increasing consumption of energy, we should consider energy consumption as a major factor to examine its impact on the production process. Only when energy consumption is taken as the main production influencing factor can it truly reflect the coordinated relationship between energy consumption and economic growth.

Therefore, this article introduces the Cobb Douglas production function and adds the energy index into it as a major factor. Through the improved Cobb-Douglas two-factor production function model, the potential relationship between economic growth and energy consumption is explored through the multivariate co-integration test on the panel data of China from 1985 to 2018.

The contribution of our empirical research is twofold. First of all, in order to remedy the econometric problem of false regression caused by the omission of related variables, this study adopts a multivariate causality test to incorporate energy variables into Cobb Douglas production function. In this way, capital and Labour variables are incorporated into the model between energy consumption and economic growth. Secondly, this study adopted VEC method of cointegration, because it can distinguish long-term and short-term relationships between variables, and can effectively identify causality Source of co-integration relationship. Then, the impulse response function is used to explain and analyze the fluctuation degree of the impact of energy consumption on economic growth. In this way, it will be possible to produce more accurate and reliable results. As a result, it provides feasible policy recommendations for energy use and economic development.

The rest of this article is organized as follows: Section 2 analyzes the current situation of China’s economic growth trend and the contradiction between energy supply and demand; Section 3 describes the improved Cobb-Douglas two-factor production function model and data; Section 4 introduces the unit root test Results, VEC co-integration test results, impulse response function analysis results, and then conduct policy analysis in Section 5, and finally draw conclusions.

## 2. Analysis of economic growth and energy structure

### 2.1. Scale and trend of economic growth

**Fig 1:**
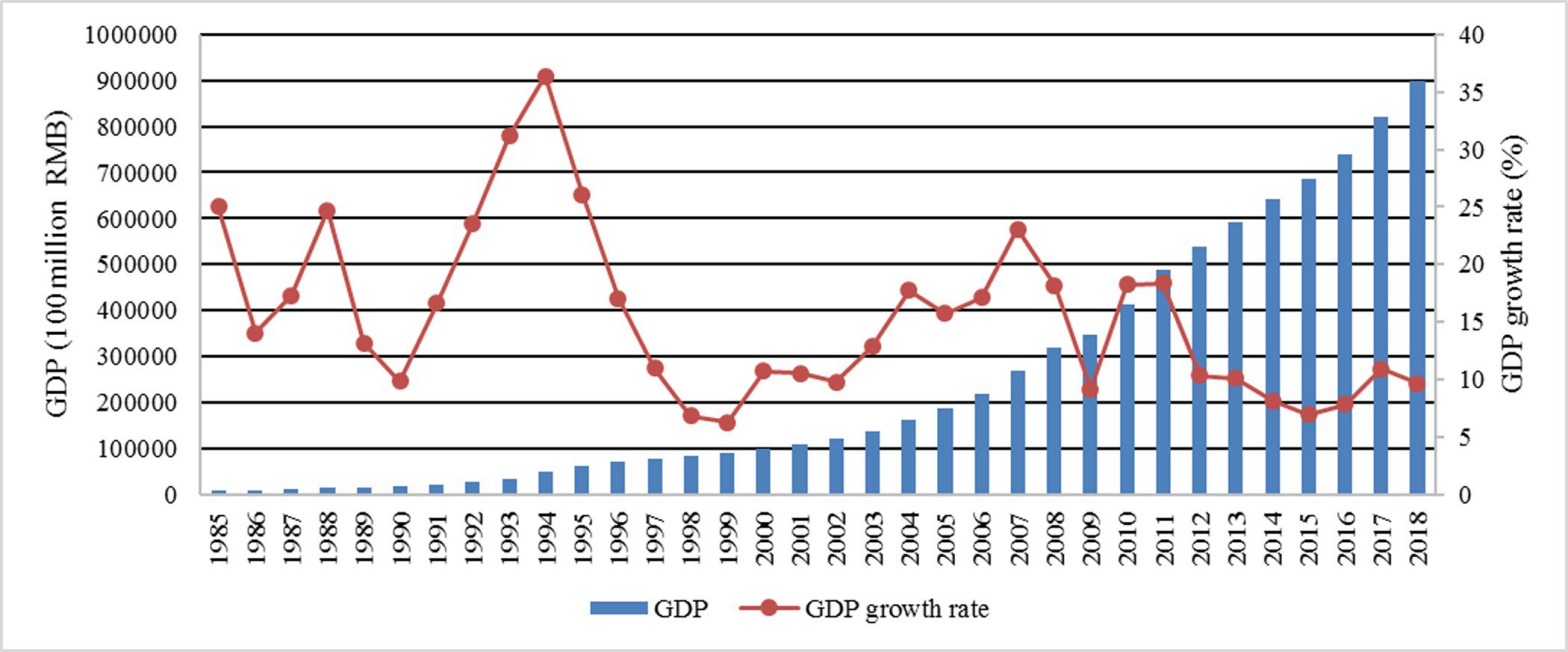
GDP and its growth rate from 1985 to 2018. Data source: China Statistical Yearbook. https://data.stats.gov.cn/search.htm?s=GDP.

As can be seen from Fig 1, From the 1985 to the present, China’s economic operation has appeared two cycles. The first economic cycle was from 1985 to 1999. Driven by reform and opening up, China’s economy developed rapidly and its overall national strength grew rapidly. After the peak in 1996, China returned to the trough of economic growth of 7.6% in 1999. The second cycle began in 1999. The consecutive five-year growth from 2003 to 2007 brought the 2007 GDP growth to a peak of 11.9%, and then gradually fell back amidst volatility. From the perspective of the overall GDP, China’s GDP will eventually flatten out after a rapid growth, and the economy will maintain a high level of development.

### 2.2. Energy structure of supply and demand

**Fig 2:**
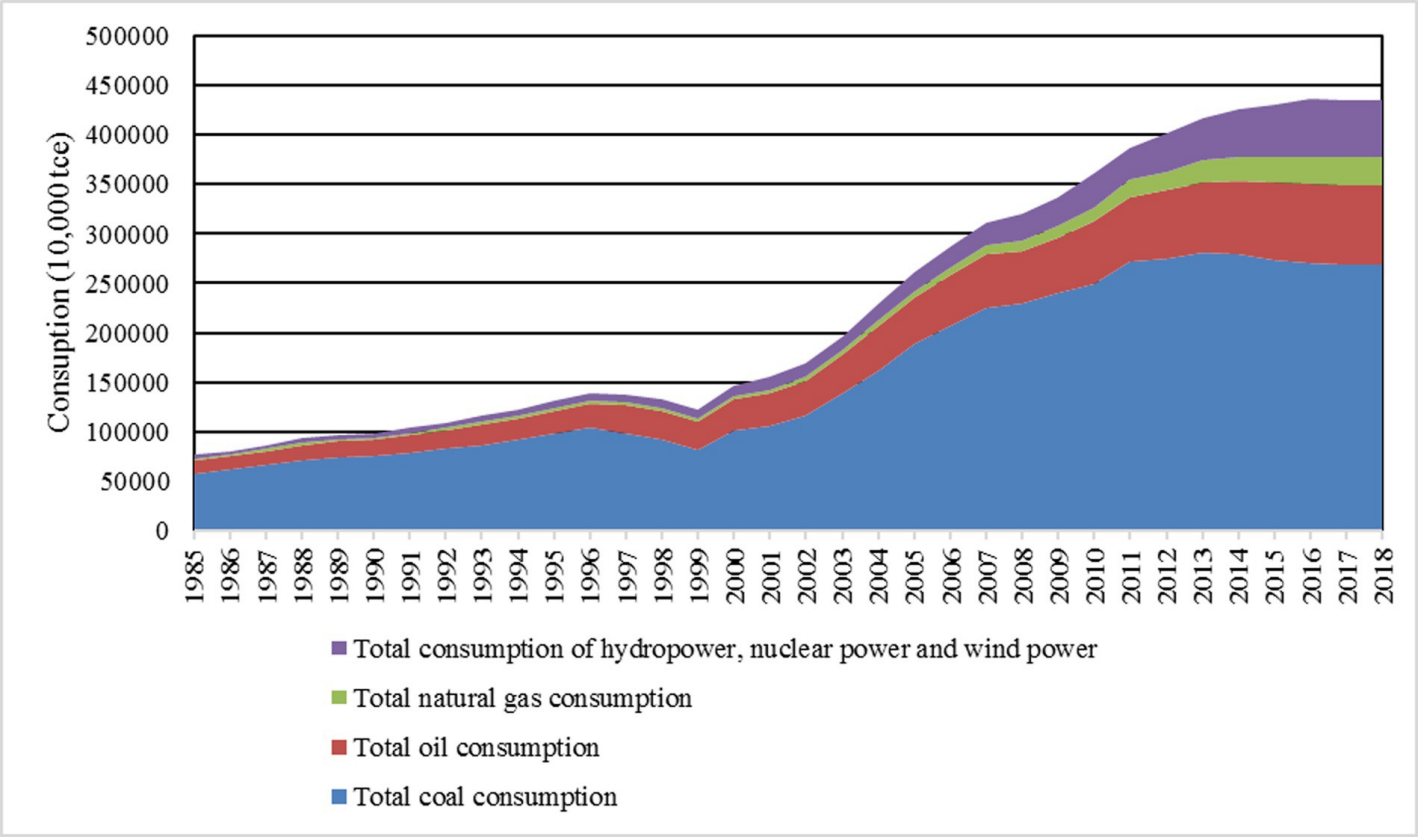
China’s total energy consumption and its composition from 1985 to 2018.

As can be seen from Fig 2, China’s total energy consumption in 1985 was 766.83 million tons of standard coal equivalent (tce). By 2016, the figure had increased to 4358.1792 million tce, which was approximately 5.68 times that of 1985. During this period, 1985–1996 was a period of steady growth. However, the total energy consumption declined from 1996 to 1999 and started to rise in 1999. The total energy consumption increased rapidly from 2001 to 2013, and then slowed down.

**Fig 3:**
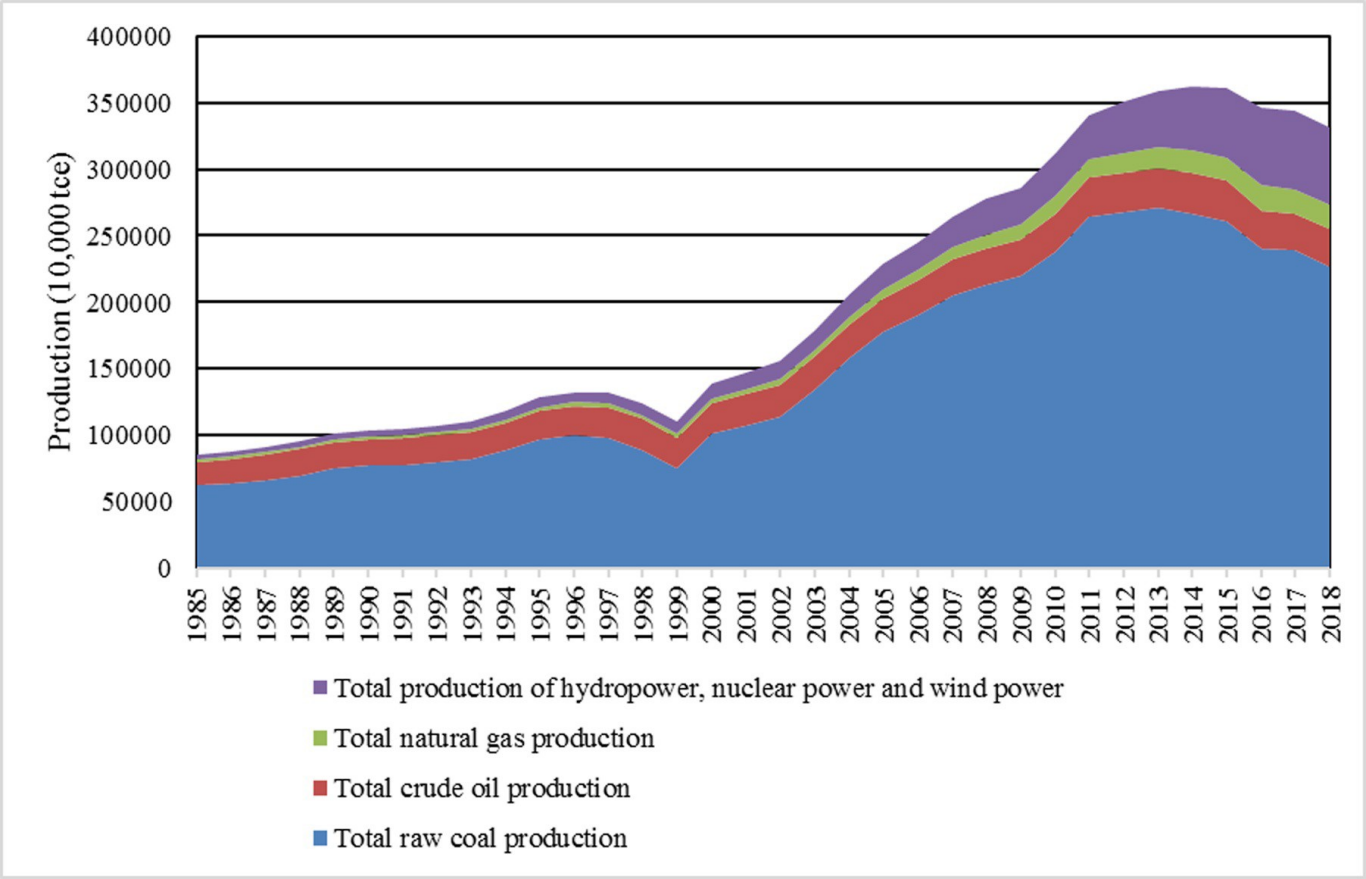
China’s total energy production and its composition from 1985 to 2018. Data source: China Statistical Yearbook. https://data.stats.gov.cn/index.htm.

As can be seen from Fig 3, China’s raw coal output has always accounted for a large proportion in the total energy production, followed by the output of crude oil. It was not until 2009 that the output of hydro power, nuclear power and wind power exceeded that of crude oil. In terms of coal output, China produced 622.7 tons of standard coal in 1985, but the figure rose to 2481.6 million tons in 2016, an increase of nearly 3.87 times. However, from 2016 to 2018, the output of raw coal fell under the environmental pressure of energy conservation and emission reduction, while the output of water, nuclear and wind power rose significantly.

Through the comparison of Figs 2 and 3, the difference between energy production and consumption structure changes appeared in 2016. The main reason is that under the environmental protection pressure of energy conservation and emission reduction, China’s coal production and consumption have been reduced, while clean energy has been greatly developed. Of course, the development and popularization of new energy technologies are still in their infancy, and energy supply is under increasing pressure.

**Fig 4:**
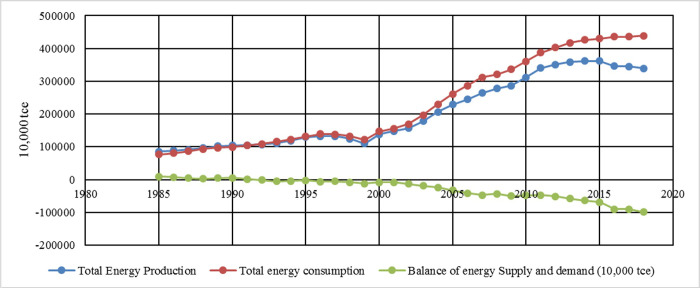
Changes in China’s energy supply and demand from 1985 to 2018.

As can be seen from Fig 4, in terms of production and consumption, China’s total energy consumption was lower than the total energy production from 1985 to 1991. While since 1991, the total energy consumption has been higher than the total energy production, and the demand is greater than the supply. Especially after 2002, the gap between the growth rate of total energy production and total energy consumption has been widening, leading to a continuous expansion of the gap between energy supply and demand. By 2018, the gap between total energy production and total energy consumption had reached 900 million tons of standard coal.

The uncoordinated development between China’s energy and economy is deepening. Under the pressure of low carbon emission, the coal-dependent energy structure must be transformed to adapt to the economic and ecological development. In terms of economic development, an economic transition from the supply side to the consumption side must also be made to adapt to the pressure of short-term clean energy supply.

### 2.3. Coordinated development between energy and economy

**Fig 5:**
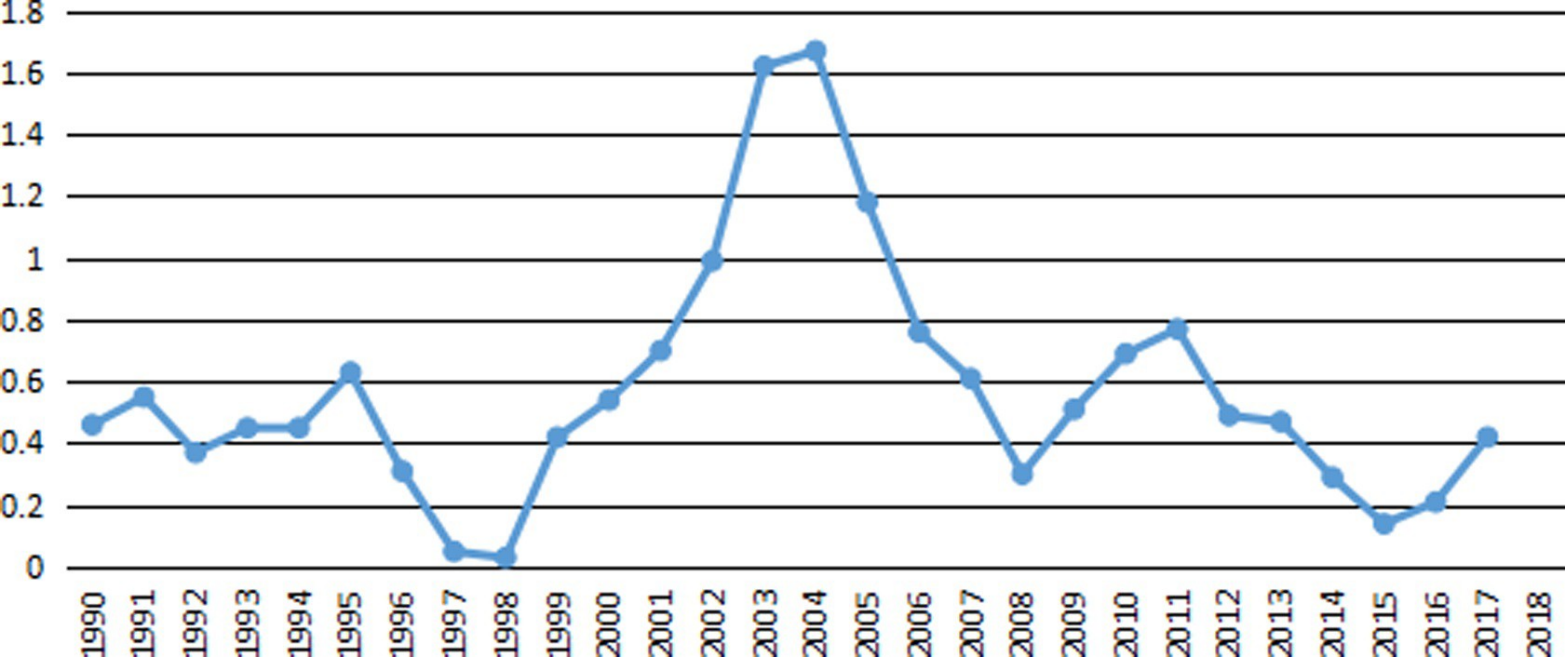
China’s energy elasticity consumption coefficient from 1990 to 2018.

Energy consumption elasticity coefficient belongs to the acceleration ratio (energy consumption elasticity coefficient = annual growth rate of energy consumption/annual growth rate of economy), which is mainly used to measure the change of energy consumption required by unit economic growth. To some extent, it reflects energy efficiency. The trend of China’s energy consumption elasticity coefficient from 1990 to 2018 is shown in Fig 5.

From Fig 5, it can be seen that from 1990 to 2018, except for 2002, 2003 and 2004, the overall elasticity coefficient of energy consumption is less than 1, which indicates that the growth rate of energy consumption in most years from 1990 to 2018 is slower than that of economic growth. And the situation greater than 1 did not appear again after 2004, suggesting that energy efficiency has risen since then. In general, the elasticity coefficient of energy consumption is generally less than 1, indicating that the economic growth rate during this period is faster than the growth rate of energy consumption.

## 3. Methodology and data

### 3.1. Cointegration

Definition 1: There is (*d*, *b*)-order cointegration between the components of the k-dimensional vector *Y* = (*y*_1_, *y*_2_, …, *y*_*k*_)’, denoted as *Y* ~ *CI* (*d*, *b*), if it is satisfied:

- y_1_, y_2_, …, y_*k*_ are all single integers of order *d*, that is, *y*_i_ ~*I*(*d*), *i* = 1, 2,…, *k*, requiring each component of *Y*: *y*_i_ ~*I* (*d*);
- There is a non-zero vector *β* = (*β*_1_, *β*_2_,…, *β*_*k*_), so that *β’ Y~I(d-b)*, *0<b≤d*.

*Y* is referred cointegrated, vector *β* is also called cointegrating vectors.

There are two main testing methods for cointegration, one is the EG two-step method, the other is the Johansen test.

#### (1) EG two-step method

If *k*+1 sequences *y* and *x*_1_, *x*_2_,…, *x*_*k*_ are all single integer sequences of order 1, then a regression equation is established for them:

$$y_{t}=X_{t}\beta +u_{t},$$ (1)

where, *y*_*t*_ is the dependent variable, *X*_*t*_ = (*x*_1*t*_, *x*_2*t*_, …, *x*_*kt*_) is the vector of explanatory variables, *β* is the *k*-dimensional coefficient vector, and *u*_*t*_ is the disturbance term.

The residual estimated by the model (1) is

$${\hat{u}}_{t}=y_{t}-{\hat{\beta }}_{1}x_{1t}-{\hat{\beta }}_{2}x_{2t}-\cdots -{\hat{\beta }}_{k}x_{kt}.$$ (2)

EG or AEG test is carried out to check whether the residual sequence *û*_*t*_ is stable. If it is stable, it indicates that it has a cointegration relationship; otherwise, it does not.

#### (2) Johansen test

The Johansen test consists of two test methods, one is trace test, and the other is maximum characteristic root test. The null hypotheses of these two test methods are different, and the test statistics used are different.

### 3.2. Cointegration test model

Given the weaknesses related to the dual causal relationship framework, this study implements the multi-causal relationship framework by incorporating capital and labor variables into the model between energy consumption and economic growth based on the neoclassical collective production theory. The relationship between the quantity of various production factors used in the production process and the maximum output that can be produced is often expressed by economists as a production function. The Cobb-Douglas two-factor production function model (C-D model) was first proposed by C.W. Cobb and Paul H. Douglas in 1928 [4–6]:

$$Y=AK^{\alpha }L^{\beta },$$ (3)

where, *A* represents the comprehensive technical level, *K* represents capital, *L* represents labor, *α* represents the output elasticity of capital (0*<α<*1), and *β* represents the output elasticity of labor (0*<β<*1). If *α+β>*1, then it means that the return to scale is increasing. If *α+β<*1, then it means that the return to scale is diminishing, that is, according to all current technical conditions, it is not cost-effective to expand the scale of production in order to increase output. If *α+β =* 1, then the return to scale is constant.

Although C-D model is widely used in economic theoretical research, we can see from the Eq (3) that this model only explains the impact of capital and labor on the economy, and does not take into account the energy as a major role in the process of economic growth [42–47]. In the early years, Wang, Y. and Zhou, J. et al. introduced energy consumption as the main influence factor into the Koserglas production function to examine the relationship between energy consumption and economic growth [48]. Later, many scholars improved the production function with energy consumption, and some scholars applied it in the microeconomic evaluation [49–52].

Therefore, this paper further expands the C-D model of the Eq (2). The explained variable is China’s GDP, and the explanatory variable is the amount of fixed asset investment, employees and the total energy consumption. Among them, fixed asset investment quantity is used to replace capital input quantity, and employment quantity is used to replace labor input quantity. The newly developed autoregressive distributed lag (ARDL) boundary test method is used to study the long-term equilibrium cointegration relationship between economic growth and explanatory variables: capital (K), labor employment (L) and energy consumption (E). Thus, we can get the three-factor production function model represented by Eq (4):

$$Y_{t}=f\left(K_{t},\;L_{t},\;E_{t}\right)\Leftrightarrow GDP_{t}=AK_{t}^{\alpha }L_{t}^{\beta }E_{t}^{\gamma }.$$ (4)

By taking the logarithmic form of Eq (4), we can get:

$$LGDP_{t}=a_{0}+\alpha LK_{t}+\beta LL_{t}+\gamma LE_{t}+\varepsilon_{t},$$ (5)

where, the logarithmic form of the variable means that the variable now exists as a growth rate. Coefficients *α*, *β*, and *γ* respectively refer to the elasticity coefficients of capital (K), labor employment(L), and total energy consumption (E).

Once the order of integration is determined, the ARDL boundary test method is used to check whether there is a long-term equilibrium relationship between the variables. ARDL model is a general dynamic specification that uses the simultaneous lag values of dependent variables and independent variables to directly estimate short-term effects, and indirectly estimate long-term equilibrium relationships. The ARDL technique uses the following unrestricted error correction model (UECM) to estimate the existence of long-term relationships:

$$\begin{array}{l} d\ln GDP_{t}=c_{0}+\sum_{i=1}^{n}a_{i}d\ln GDP_{t-i}+\sum_{i=1}^{n}\alpha_{i}d\ln K_{t-i}+\sum_{i=1}^{n}\beta_{i}d\ln L_{t-i}+\sum_{i=1}^{n}\gamma_{i}d\ln E_{t-i}\\ \;+\lambda_{1}\ln GDP_{t-1}+\lambda_{2}\ln K_{t-1}+\lambda_{3}\ln L_{t-1}+\lambda_{4}\ln E_{t-1}+\mu_{t}, \end{array}$$ (6)

where, *d* is the first difference operator, *μ*_*t*_ is the white noise error term, *n* is the number of years, lnGDP is the natural logarithm of real GDP, lnK is the natural logarithm of capital stock, lnL is the natural logarithm of labor employment, and lnE is the natural logarithm of energy consumption. Parameters *a*, *α*, *β* and *γ* are short-term coefficients, while *λ*_*j*_, *j* = 1,2,3,4 is the corresponding long-term multiplier of the underlying ARDL model. By testing the significance of variable lag levels, the F test is used to determine whether there is an integration relationship between variables.

In the presence of this long-term relationship, the Granger causality test can be tested by the following multivariate *q*th-order vector error correction model (VECM). Under the condition that the series is cointegrated, a lagging error correction item is added:

$$\begin{array}{l} d\ln GDP_{t}=a_{0}+\sum_{i=1}^{q}a_{1i}d\ln GDP_{t-i}+\sum_{i=1}^{q}a_{2i}d\ln K_{t-i}+\sum_{i=1}^{q}a_{3i}d\ln L_{t-i}+\sum_{i=1}^{q}a_{4i}d\ln E_{t-i}\\ \;+a_{5i}ECT_{t-1}+\mu_{1t} \end{array}$$ (7)

$$\begin{array}{l} d\ln K_{t}=\alpha_{0}+\sum_{i=1}^{q}\alpha_{1i}d\ln K_{t-i}+\sum_{i=1}^{q}\alpha_{2i}d\ln GDP_{t-i}+\sum_{i=1}^{q}\alpha_{3i}d\ln L_{t-i}+\sum_{i=1}^{q}\alpha_{4i}d\ln E_{t-i}\\ \;+\alpha_{5i}ECT_{t-1}+\mu_{2t} \end{array}$$ (8)

$$\begin{array}{l} d\ln L_{t}=\beta_{0}+\sum_{i=1}^{q}a_{1i}d\ln L_{t-i}+\sum_{i=1}^{q}\beta_{2i}d\ln GDP_{t-i}+\sum_{i=1}^{q}\beta_{3i}d\ln K_{t-i}+\sum_{i=1}^{q}\beta_{4i}d\ln E_{t-i}\\ \;+\beta_{5i}ECT_{t-1}+\mu_{3t} \end{array}$$ (9)

$$\begin{array}{l} d\ln E_{t}=\gamma_{0}+\sum_{i=1}^{q}\gamma_{1i}d\ln E_{t-i}+\sum_{i=1}^{q}\gamma_{2i}d\ln GDP_{t-i}+\sum_{i=1}^{q}\gamma_{3i}d\ln K_{t-i}+\sum_{i=1}^{q}\gamma_{4i}d\ln L_{t-i}\\ \;+\gamma_{5i}ECT_{t-1}+\mu_{4t} \end{array}$$ (10)

among them, *a*_*s*_, *α*_*s*_, *β*_*s*_, and *γ*_*s*_, *s* = 0, 1*i*, 2*i*, 3*i*, 4*i*, 5*i* are parameters to be estimated, *μ*_*t*_ is a continuous uncorrelated error item, and *ECT*_*t*-1_ is an error correction item (ECT). The F-statistics of the ECM lagging explanatory variables show the significance of short-term causal effects. The t-statistic of the lag error correction term coefficient shows the significance of the long-term causal effect. The lag length *q* is based on Schwarz Information Criteria (SIC) and/or Akike Information Criteria (AIC).

### 3.3. Data description

This article uses China’s 34 years data from 1985 to 2018, which were collected and compiled in the "China Statistical Yearbook" from 1985 to 2018. The four variables selected in this paper are: GDP (gross domestic product), K (fixed asset investment), L (total number of employees) and E (total energy consumption). In this case, since the GDP data we find from the China Statistical Yearbook are calculated in current year prices, we need to exclude the effect of price changes to give a more accurate actual GDP changes. So we treat GDP as follows:

RealGDP=nominalGDP/CPIbasedon1985. (11)

Thus, GDP based on 1985 as the base year is obtained. Like GDP, fixed asset investment also needs to exclude the effects of price changes to obtain the actual value of fixed asset investment in the corresponding year.

In order to eliminate hetero covariance, we performed the natural logarithm transformation on the variable. Of course, taking the logarithm would not change the co-integration relationship between the data. Therefore, we respectively used LGDP, LK, LL and LE to represent the natural logarithm values of GDP, fixed asset investment, total number of employees and total energy consumption.

## 4. Results and discussion

### 4.1. Unit root test

**Fig 6:**
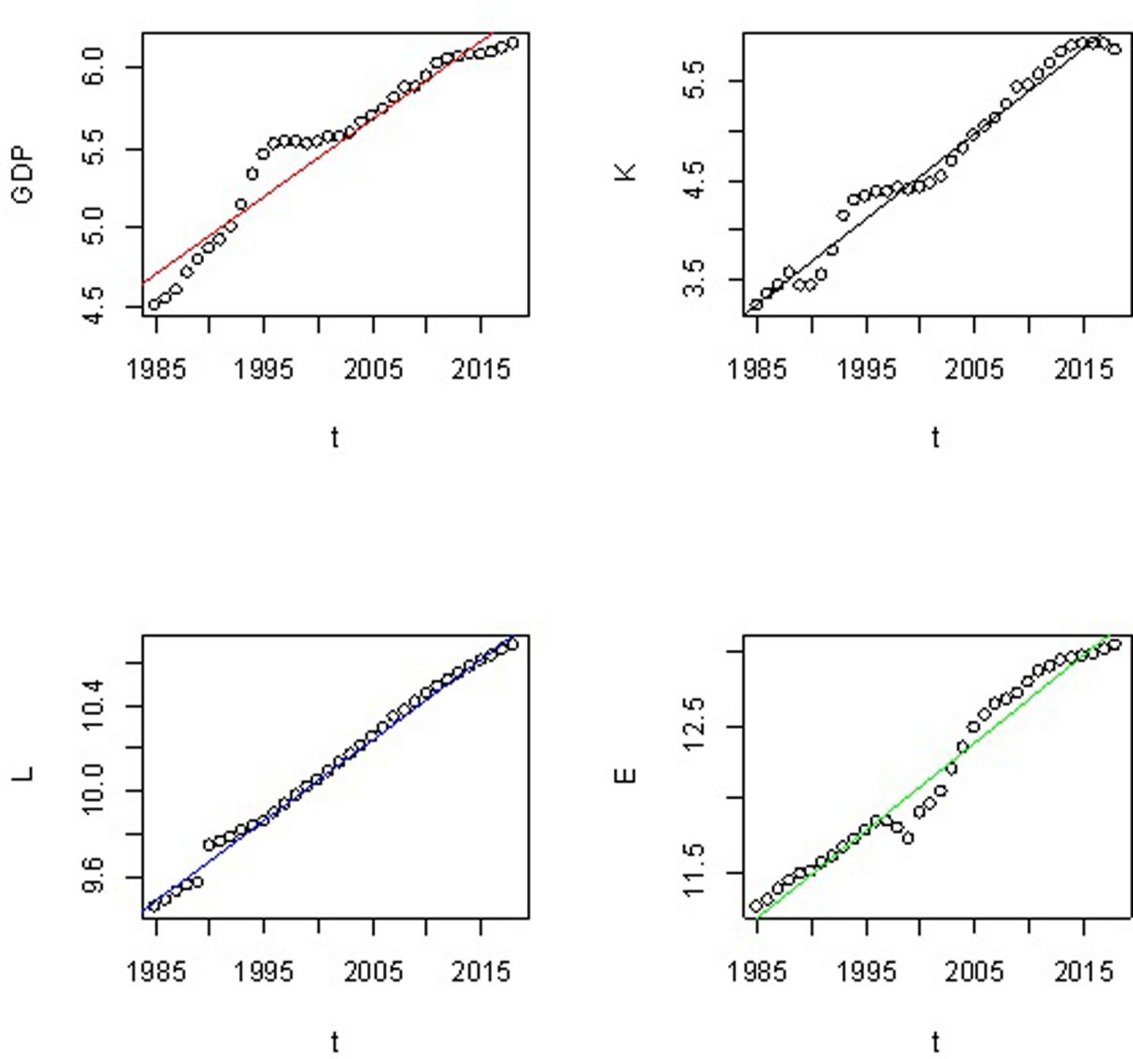
Logarithmic sequence trend chart of GDP, capital (K), labor (L), and energy (E).

As can be seen from Fig 6, the sequences of LGDP, LK, LL and LE all show a relatively similar growth trend over time. Therefore, based on this similar growth trend among them, we can guess: GDP may have a long-term co-integration relationship with capital input, labor and energy consumption. Below we will verify this conjecture through empirical analysis.

First of all, we need to conduct unit root test on the stationary state of variables. It is well known that when the variable is unstable in the regression, it is easy to cause false regression, which makes the regression results we generally use invalid. In order to obtain robust results, we used the Augmented Dickey-Fuller (ADF) unit root test and Phillips–Perron (PP) test to test the level value, first-order difference and second-order difference of each variable from 1985 to 2018. The unit root test is also very sensitive to different hysteresis structures. Therefore, we adopted the Schwartz Information Criterion (SIC) to determine the lag term. The detailed results are shown in Table 1.

**Table 1:** Unit root test results of variables.

variable	ADF test value	PP test value
Level (intercept and trend)		
LGDP	-1.991312	-3.568379
LK	-3.121304	-3.557759
LL	-1.318489	-3.587527*
LE	-1.889610	-3.557759*
First difference (intercept, no trend)		
dLGDP	-2.406947	-2.957110*
dLK	-2.915751	-2.957110*
dLL	0.420850*	-2.976263**
dLE	-2.359549*	-2.846654**
Second difference (intercept, no trend)		
ddLGDP	-5.108669*	-2.960411**
ddLK	-5.904405*	-2.960411**
ddLL	-10.372500**	-2.976263**
ddLE	-3.732632***	-2.957110***

The Schwarz Information Criterion (SIC) is used to select the optimal lag length of the variables in the ADF test equation. Use the Newey-West Bartlett kernel method to select the bandwidth of the PP test.

* Indicates significance at 10% level.

** Indicates significance at 5% level.

*** Indicates significance at 1% level.

It can be seen from Table 1 that the four data vectors LGDP, LK, LL and LE are all non-stationary sequences. Some conflicting results about the stationarity in the first difference were found in the ADF test and PP test. However, at the critical level of 10%, 5% or more stringent 1%, after taking the second difference of the variable, stationarity is found. It can be seen that they are second-order single integration, which satisfies the condition of co-integration test, and can trim them into second-order single integers to meet the conditions of cointegration test.

Therefore, we can consider the difference processing of the original sequence variables. However, since the data after difference is difficult to explain its meaning, we consider the co-integration analysis and consider the modified VEC model.

### 4.2. Cointegration test

The cointegration relationship between variable sequences was first proposed by Engel and Granger. Since false regression is likely to occur when a variable is non-stationary, the cointegration test is used to test the long-term relationship between variables. The cointegration test means that when the variable series are in an unstable state, but the linear combination of the non-stationary series is a stationary variable, it can be considered that there is a long-term stable relationship between these variables. Two-step Engel-Granger (EG) method and Johansen test are commonly used in series cointegration test [32, 53–55]. Generally, the EG two-step method can be used for the cointegration test between two variables, and the Johansson test can be used for the cointegration relationship between multiple variables. Moreover, the EG two-step method is a cointegration test based on regression residuals, and the Johansen test is a cointegration test based on regression coefficients.

#### (1) Determine the lag order

First, an unconstrained VAR model was constructed, and select the maximum 5-period lag order for testing.

As shown in Table 2, combining the judgment results of AIC and SIC, when the lag order is 5, the most information criterion can be met. So choose 5 as the maximum lag order of unconstrained VAR. Therefore, the lag order of Johansen test is 4.

**Table 2:** Determine the lag order.

Lag order	Log likelihood	AIC	SIC	F-test statistic
0	102.243130	-6.775388	-6.586796	-6.716323
1	245.347719	-15.541220	-14.598260	-15.245900
2	285.511675	-17.207700	-15.510370	-16.676120
3	297.398503	-16.924030	-14.472330	-16.156190
4	329.397251	-18.027400	-14.821320	-17.023290
5	378.853523	-20.334573*	-16.372428*	-19.091436*

* Denotes significance at 5% level.

#### (2) Johansen test

As shown in Table 3, trace test results show that there are three co-integration relationships between LGDP and LK, LL and LE, which can be used for VEC test. It is taken for granted that there is a long-term equilibrium in Eq (6). When the real GDP is the dependent variable, the long-term and short-term coefficients are estimated with the relevant ARDL and ECM. The ARDL model is estimated by setting the maximum lag length to 4 and using SIC to select the best lag order of the model. The final specification selected is ARDL (4, 4, 4, 0), that is, the hysteresis lengths of LGDP, LK, LL and LE are 4, 4, 4 and 0 respectively.

**Table 3:** Johansen test results.

Unrestricted Cointegration Rank Test (Trace)				
Hypothesized		Trace	5%	
cointegration equations	Eigenvalue	Statistic	Critical Value	Prob.**
None *	0.85914	130.69150	47.85613	0.00000
At most 1 *	0.75983	73.85251	29.79707	0.00000
At most 2 *	0.57330	32.48631	15.49471	0.00010
At most 3 *	0.23550	7.78761	3.84147	0.00530

Trace test indicates 4 cointegrating equations at the 5% level.

* denotes rejection of the hypothesis at the 5% level.

**MacKinnon-Haug-Michelis (1999) p-values.

### 4.3. Causality test

#### (1) Error correction model

As shown in Table 3, there is a long-term co-integration relationship between energy consumption and economic growth. The positive correlation between the two may be due to the increase in energy consumption which promotes economic growth, or it may be due to the fact that economic growth drives energy consumption.

In the case of co-integration between variables, compared with the vector autoregression (VAR) method, vector error correction (VEC) model can distinguish the long-term relationship and short-term relationship between variables, and can identify the source of causality that cannot be detected by the usual Granger causality test. Therefore, vector error correction (VEC) model can be established to further clarify the correlation between them. In Table 3, we know that the lag order is 4. Therefore, in the VEC model, the lag order is set to 4, and the cointegration relation equation number is 3. So as to obtain the VEC cointegration relation equations of the time series, as shown in Table 4. The following results are obtained through Eviews10 software.

**Table 4:** Estimated long-run coefficients based on ARDL (4,4,4,0).

Regressor	Coefficient	Standard error	t-statistics [p-value]
Dependent variable: LGDP			
LK	-1.2077	0.0241	-63.2903[0.000]
LL	-0.5447	0.0179	-123.1400[0.000]
LE	-1.5309	0.0124	-43.6853[0.016]
Intercept C	12.6673	21.7786	-3.4978[0.000]

Table 4 shows the cointegration relationship with the largest log likelihood value, which is also the cointegration relationship regression in VEC. According to Table 4, the cointegration equation of this paper can be written as:

LGDP=1.2077*LK+0.5447*LL+1.5309*LE-12.6673*C. (12)

Through this co-integration relationship, we can know that both LGDP and LE are positively correlated long-term equilibrium relationships, that is, for every unit of GDP increase, total energy consumption will increase by 1.5309 units. In the VEC model, the co-integration relationship is expressed as the error correction term:

VECM=LGDP-1.2077*LK-0.5447*LL-1.5309*LE+12.6673*InterceptC. (13)

The coefficient matrix of the VEC model is shown in Table 5.

**Table 5:** Estimated coefficient matrix of VEC model.

Regressor	Coefficient	Standard error	t-statistics [p-value]
Dependent variable: dLGDP			
dLGDP(-1)	-0.30328	0.07588	1.35288[0.000]
dLGDP(-2)	-0.53446	0.57045	0.56406[0.027]
dLGDP(-3)	-0.30197	0.24320	2.57069[0.000]
dLK	-1.93079	0.83764	-0.24320[0.000]
dLK(-1)	-0.90797	0.85924	2.31930[0.000]
dLK(-2)	-0.52364	0.68992	-2.30505[0.049]
dLK(-3)	-0.13741	0.25885	0.25131[0.017]
dLL	-0.56745	0.57045	-0.25885[0.028]
dLL(-1)	-1.57452	0.47326	-0.13741[0.036]
dLL(-2)	-0.25885	0.41316	-1.88386[0.025]
dLL(-3)	-1.42302	2.31930	1.20538[0.023]
dLE	-1.34587	3.65815	0.74203[0.197]
d Intercept C	-0.41316	0.41316	-1.66985[0.0236]

According to the coefficient matrix, when LK, LL, LE remain unchanged, the change of LGDP in period t can eliminate 30.33% of the change in period t-1. When LGDP, LL, LE remain unchanged, and LK remains unchanged at the t-th LGDP, LK, LE, the change of LL in the t-th period can increase the change in t-1 by 56.41%. When LGDP, LK, and LL remain unchanged, the change of LE in period t can eliminate 134.59% of the unbalanced error in period t-1.

Using the estimated model, it can be seen that the change in energy consumption is divided into two parts: one part is due to the impact of economic development. In the short term, for every 1% increase in economic growth, energy consumption will increase by 1.53%. The other part is due to the deviation of energy consumption in the previous period from the long-term equilibrium relationship. The error correction coefficient in the model is -1.345871, which conforms to the reverse correction mechanism. It can be seen that the error correction term will converge energy consumption to the long-term equilibrium state with the adjustment force of 134.59%.

#### (2) Impulse response function

**Fig 7:**
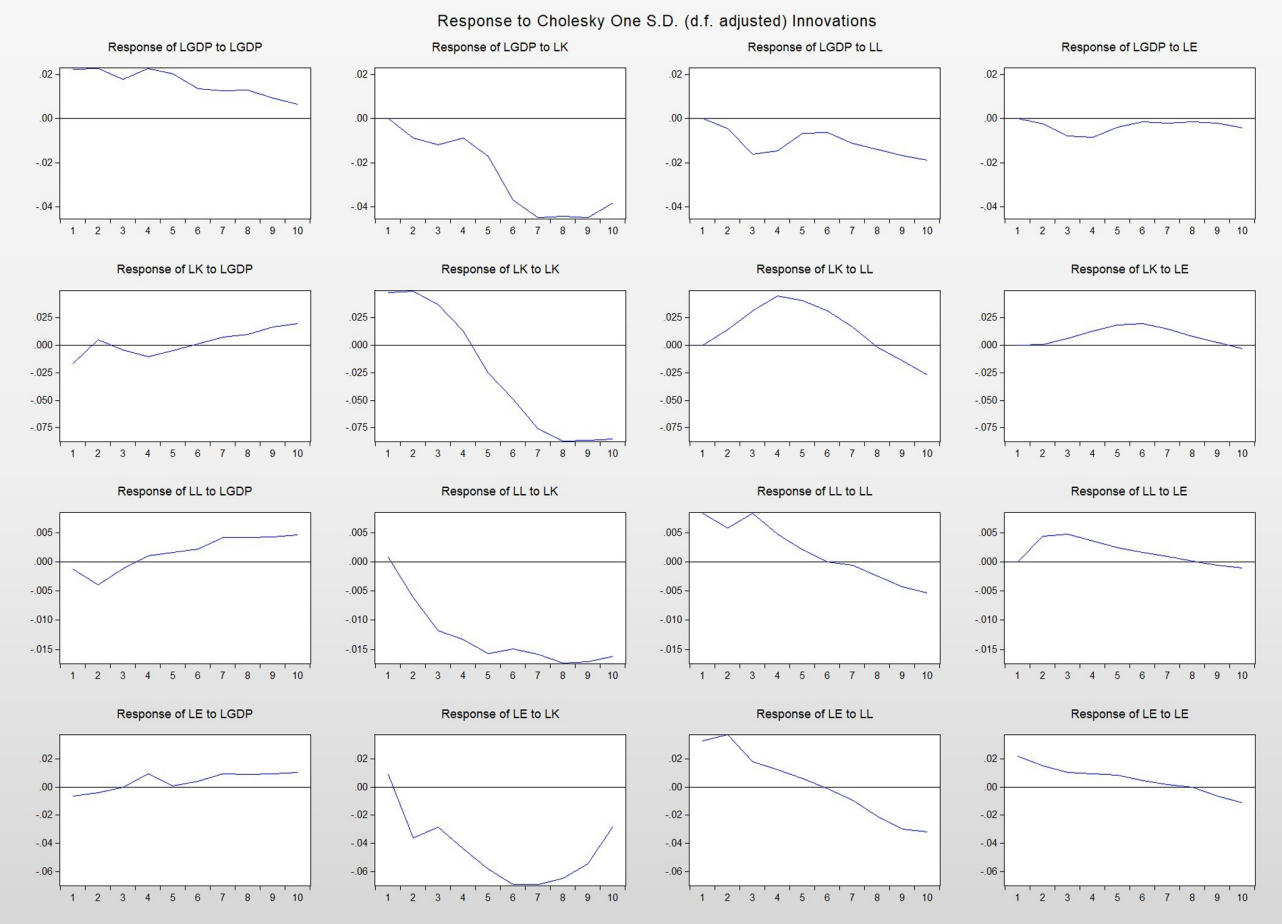
Impulse response diagram.

**Fig 8:**
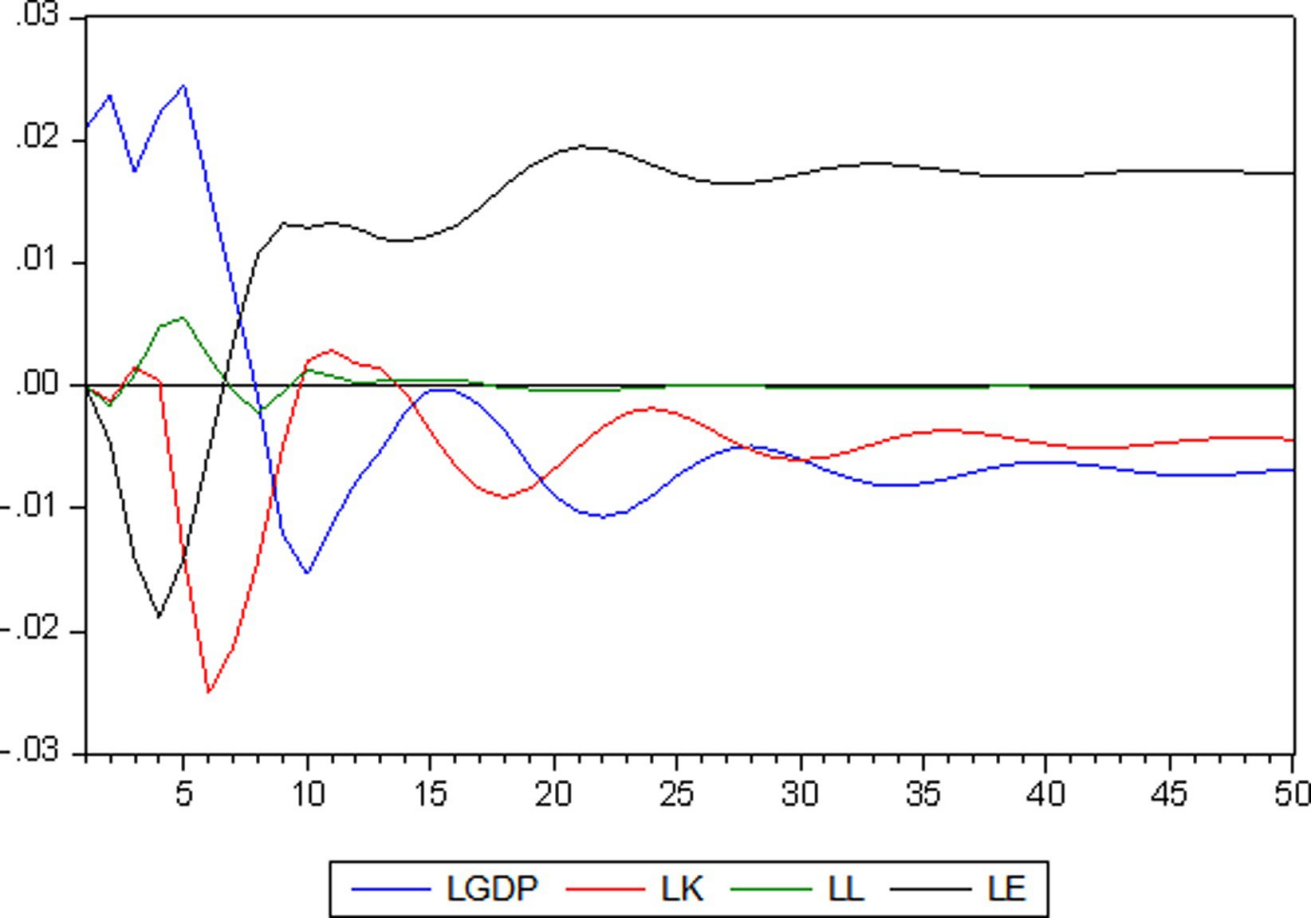
Impulse response results of LGDP affected by the four variables.

As can be seen from Figs 7 and 8, LGDP fluctuated before the seventh period and fell rapidly after the seventh period, and reached the peak of negative impact in the eleventh period. After the impact of LE, LGDP had a negative impact before the sixth phase, and reached the peak of negative impact in the fourth phase. After the fourth phase, the impact of LE on LGDP gradually increased, and turned into a positive impact after the sixth phase. This means that in the short term, we put in energy, but the economy did not grow immediately. This is also easy to explain. Energy, as a raw material, often needs to be processed, and this process takes a long time. In addition, some energy products cannot be bought or sold, and they cannot produce visible economic benefits. However, in the long run, the positive effect of energy input on economic growth is beyond doubt.

#### (3) Variance decomposition

**Fig 9:**
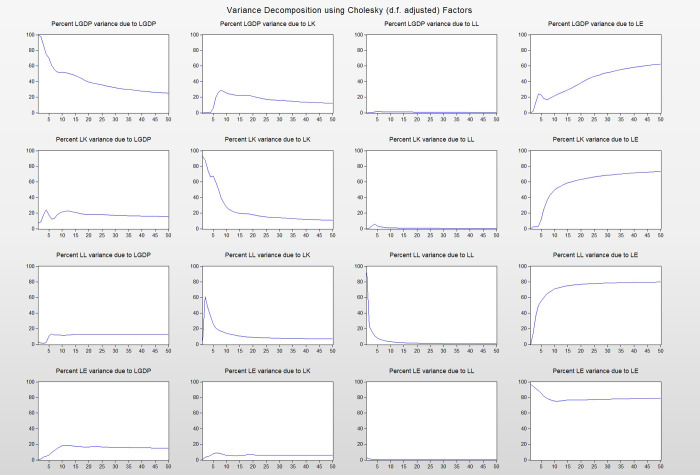
Diagram of variance decomposition.

**Fig 10:**
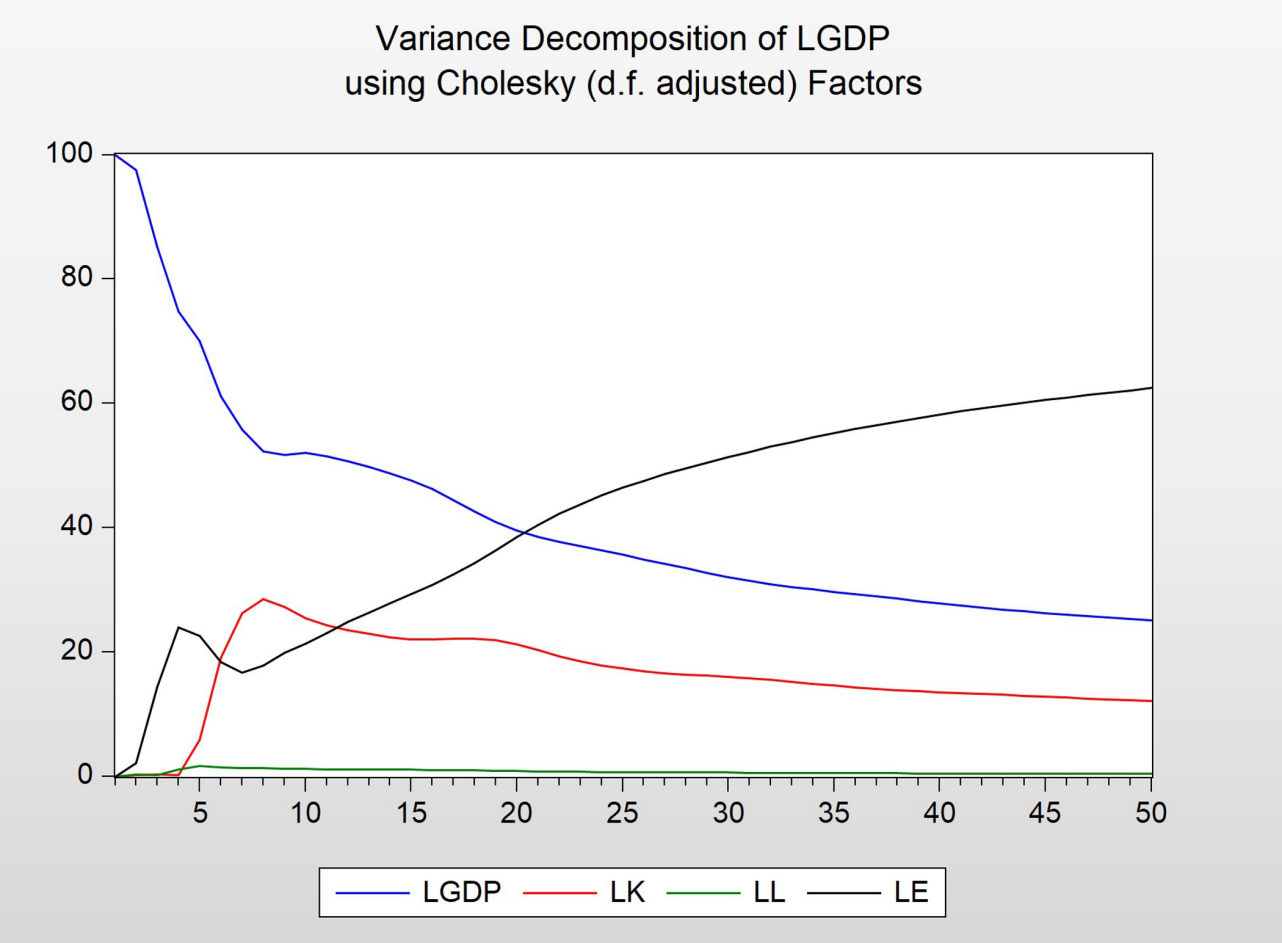
Variance decomposition results of LGDP.

The variance decomposition result of LGDP is shown in Figs 9 and 10. At the beginning, the contribution rate of LGDP to itself was in the first place. As the number of periods increases, the proportion of LGDP gradually decreases, which is explained by its own changes. The part of LGDP explained by LE is gradually increasing, and about 60% of changes in LGDP can be explained by changes in LE. The part of LGDP variance explained by LK changes reaches its maximum value in the eighth period and then gradually decreases. Finally, about 10% variance change of LGDP can be explained by LK change. In the variance change of LGDP, the amount that LL can explain is always the smallest, no more than 2%, and reaches the maximum value in the fifth period.

## 5. Policy implications

This article selects China’s GDP data, fixed capital formation, total employment and total energy consumption from 1985 to 2018 for a comprehensive analysis. we can conclude that there is a positive correlation between energy consumption and GDP: GDP growth of 1%, total energy consumption increased by 1.53%, which means that economic growth requires energy support. At the same time, the error correction term will converge energy consumption to a long-term equilibrium state with an adjustment intensity of 134.59%. From the results of variance decomposition, we can see that as the number of periods increases, the part of LGDP explained by LE gradually increases, and about 60% of the change in LGDP can be explained by the change of LE. In the short run, it should be pointed out that (1) energy input is conducive to economic development, (2) China is an energy-dependent country, and (3) negative impact on energy, such as an increase in energy prices and a reduction in energy supply or energy conservation policy shocks, may have a major adverse impact on economic growth.

However, as can be seen from the results of impulse response function and variance decomposition, in the short term, energy-saving and emission-reduction policies have a greater impact on GDP growth, but in the long term, as the number of episodes increases, the impact of energy-saving and emission-reduction policies on GDP growth is gradually exhausted and converges to the long-term equilibrium state.

It can be seen from the results of the error correction model causality t-statistic that China has gradually overcome its heavy dependence on coal by diversifying its energy supply. However, due to the negative impact on economic growth, it is currently not feasible for China to take direct measures to reduce energy consumption. Therefore, more effective policies and measures should be implemented.

First, improve energy efficiency. Low energy efficiency will result in a large amount of energy input but few finished products after processing. Therefore, we should increase scientific research and improve energy efficiency. Second, optimize the industrial structure. After 40 years of reform and opening up, the rapid development of China’s economy is inextricably linked with the development of China’s secondary industry. However, the proportion of energy consumption in China’s secondary industry is relatively large. Therefore, innovation should be integrated into the secondary industry to eliminate high pollution emissions and actively support green and efficient enterprises. The third policy option is to raise low-carbon awareness, develop new energy sources, and improve and optimize the energy structure. The focus is on the development of renewable energy such as hydropower, wind power, and solar energy, as well as clean energy such as nuclear power and natural gas.

## 6. Conclusions

Based on data from 1985 to 2018, this paper focuses on the relationship between energy consumption and economic growth in China. The main conclusions are as follows: Firstly, we learn from the co-integration test that there is a long-term equilibrium between China’s economic growth and energy consumption, that is, every 1% increase in GDP will increase energy consumption by 1.53%. Secondly, the results of impulse response and variance decomposition show that in the short term, GDP will be adversely affected by energy input, but in the long term, the impact of energy-saving and emission-reduction policies on GDP growth is gradually exhausted and converges to the long-term equilibrium state.

The empirical results of unilateral causality between energy consumption and economic growth obtained by the error correction model show that China is an energy-dependent country. Energy consumption is a key factor in economic growth. Only by diversifying energy supply, adjusting energy structure and increasing the proportion of clean energy consumption can China resolve the contradiction between pollution emission caused by energy use and economic growth.
